# Supplementary material for: Genetic Characteristics of Avian Influenza Virus Isolated from Wild Birds in South Korea, 2019–2020
Source: Viruses. 2021 Feb 27;13(3):381. doi: 10.3390/v13030381 (PMC7997295; doi:10.3390/v13030381)
Supplement: Supplementary file 1 [file viruses-13-00381-s001.pdf]

(a)

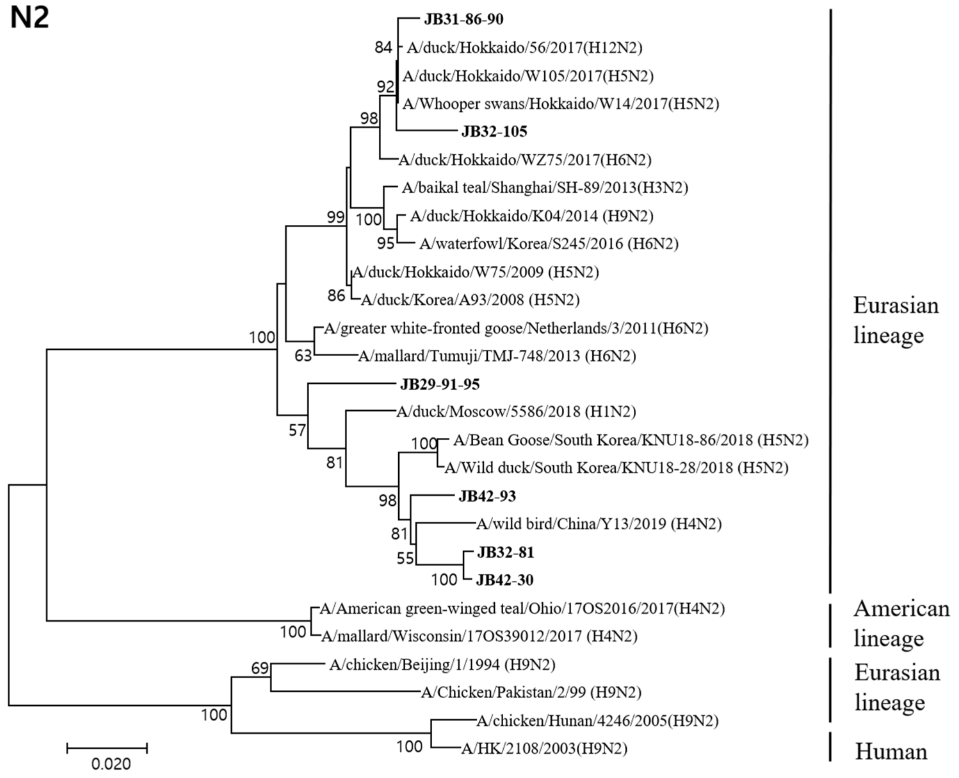

(b)

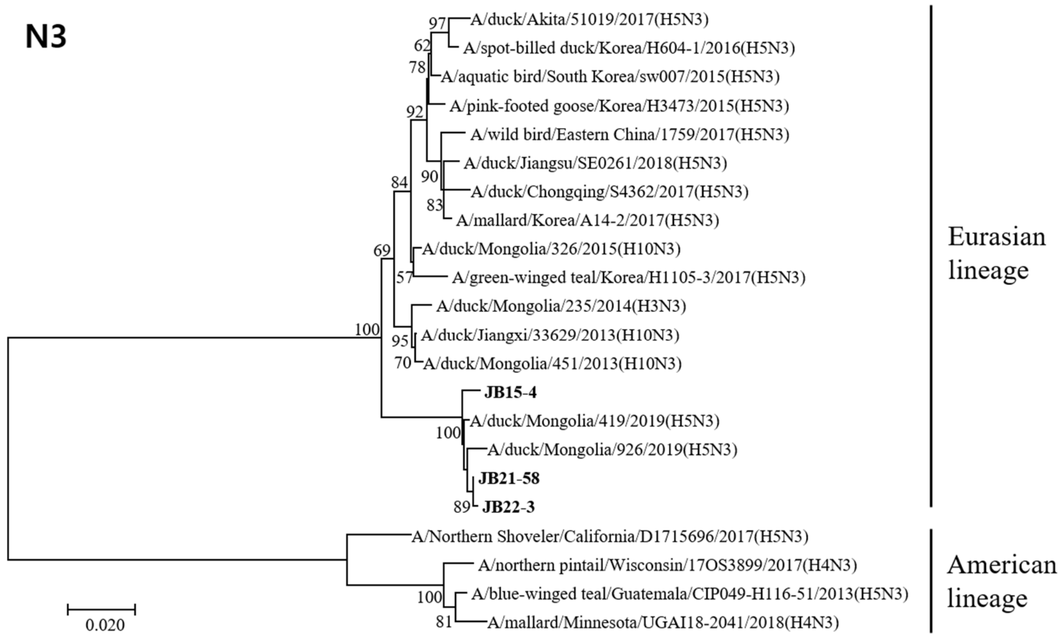

Supplementary Figure S1. Phylogenetic trees based on the nucleotide sequences of N2 (a), N3 (b), N4 (c), N6 (d), N7 (e), N8 (f) and N9 (g) genes. Trees were produced by the by neighbor-joining method and bootstrapped with 1000 replicates. Branch lengths are numbers of nucleotide substitutions per site; only bootstrap values at or above 50% are shown. Isolates identified in this study are shown in bold.

(c)

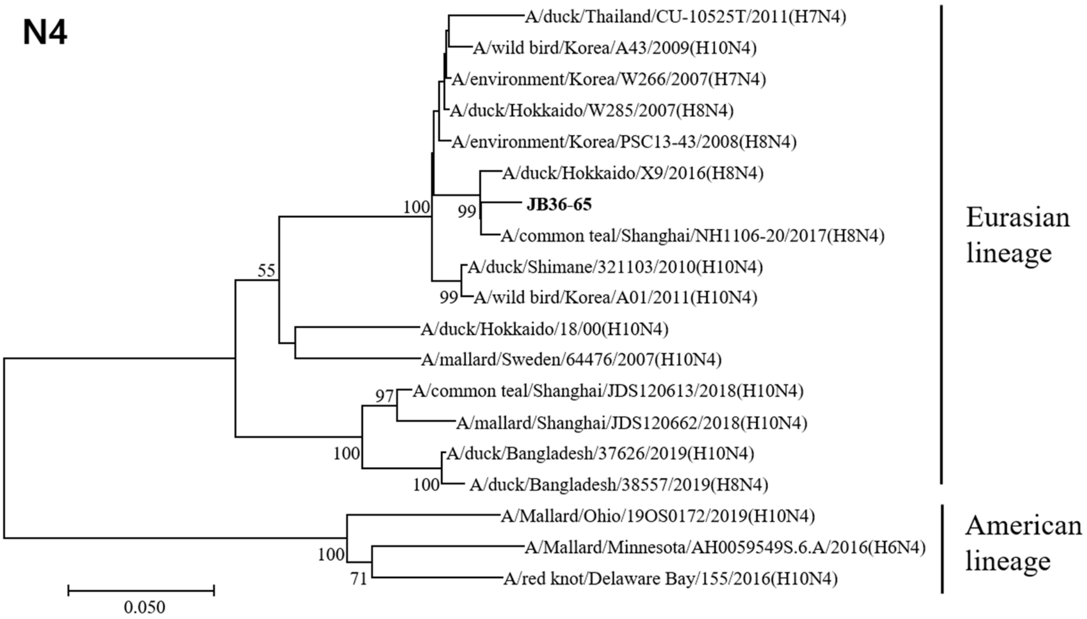

(**d**)

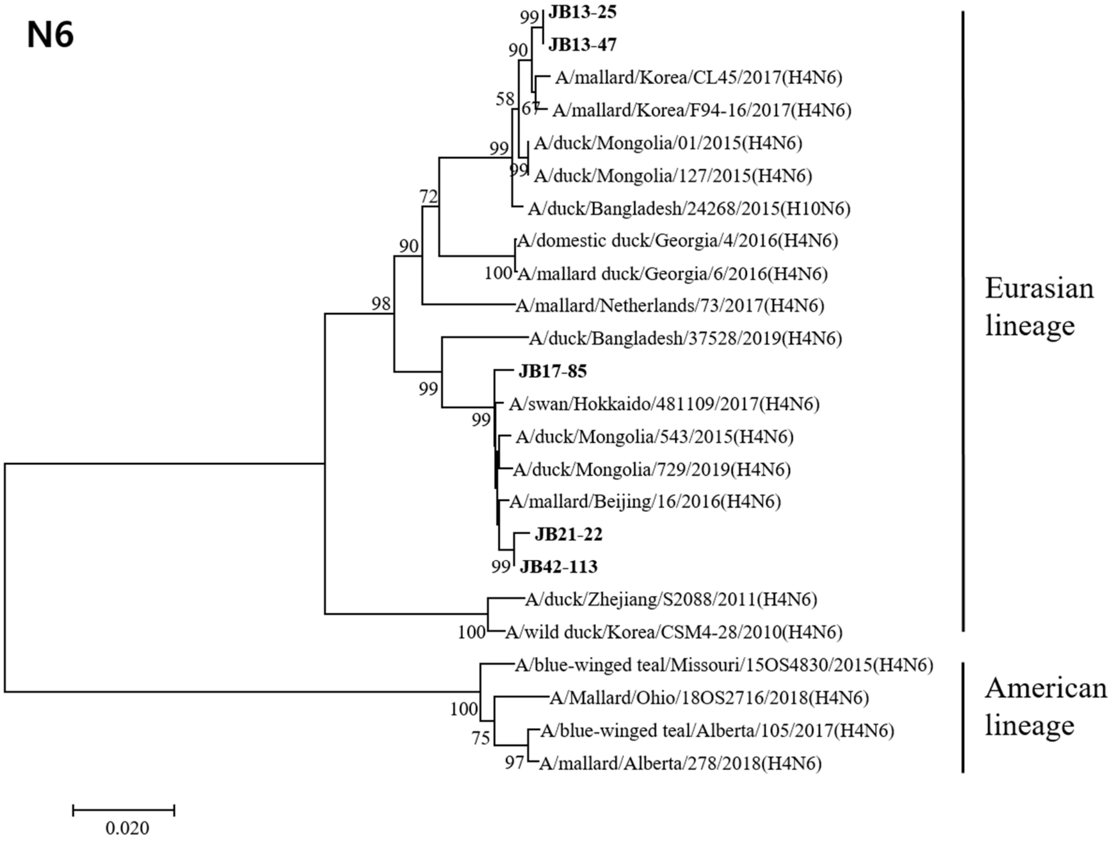

Supplementary Figure S1. *Cont.*

(e)

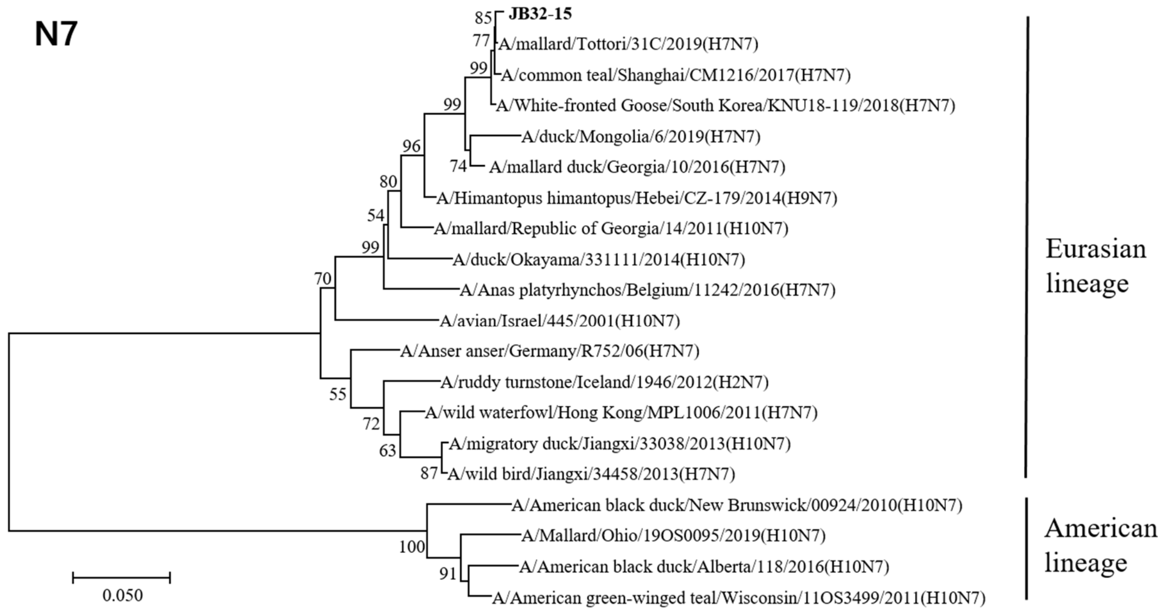

(f)

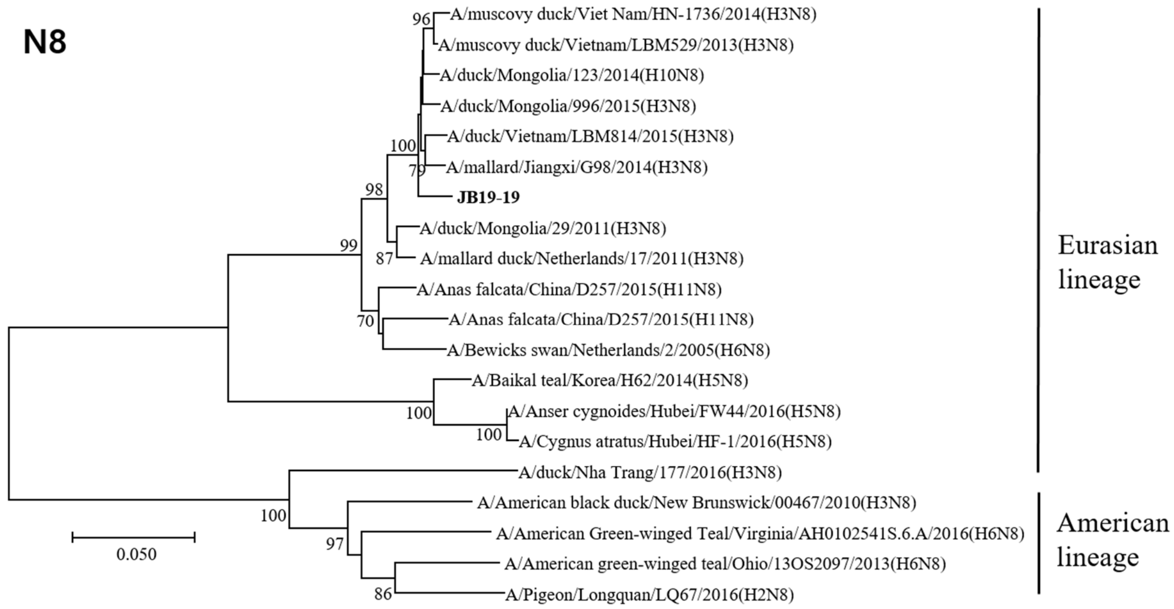

Supplementary Figure S1. *Cont.*

(g)

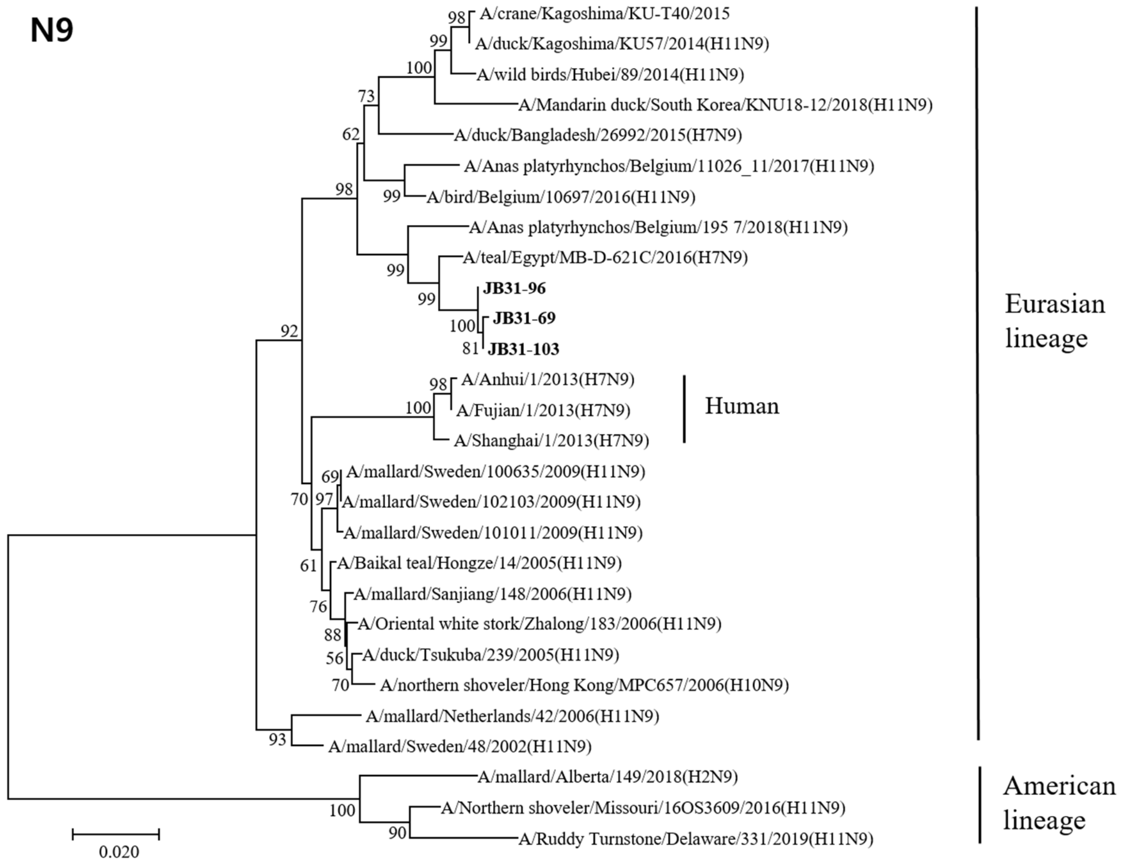

Supplementary Figure S1. *Cont.*

(a)

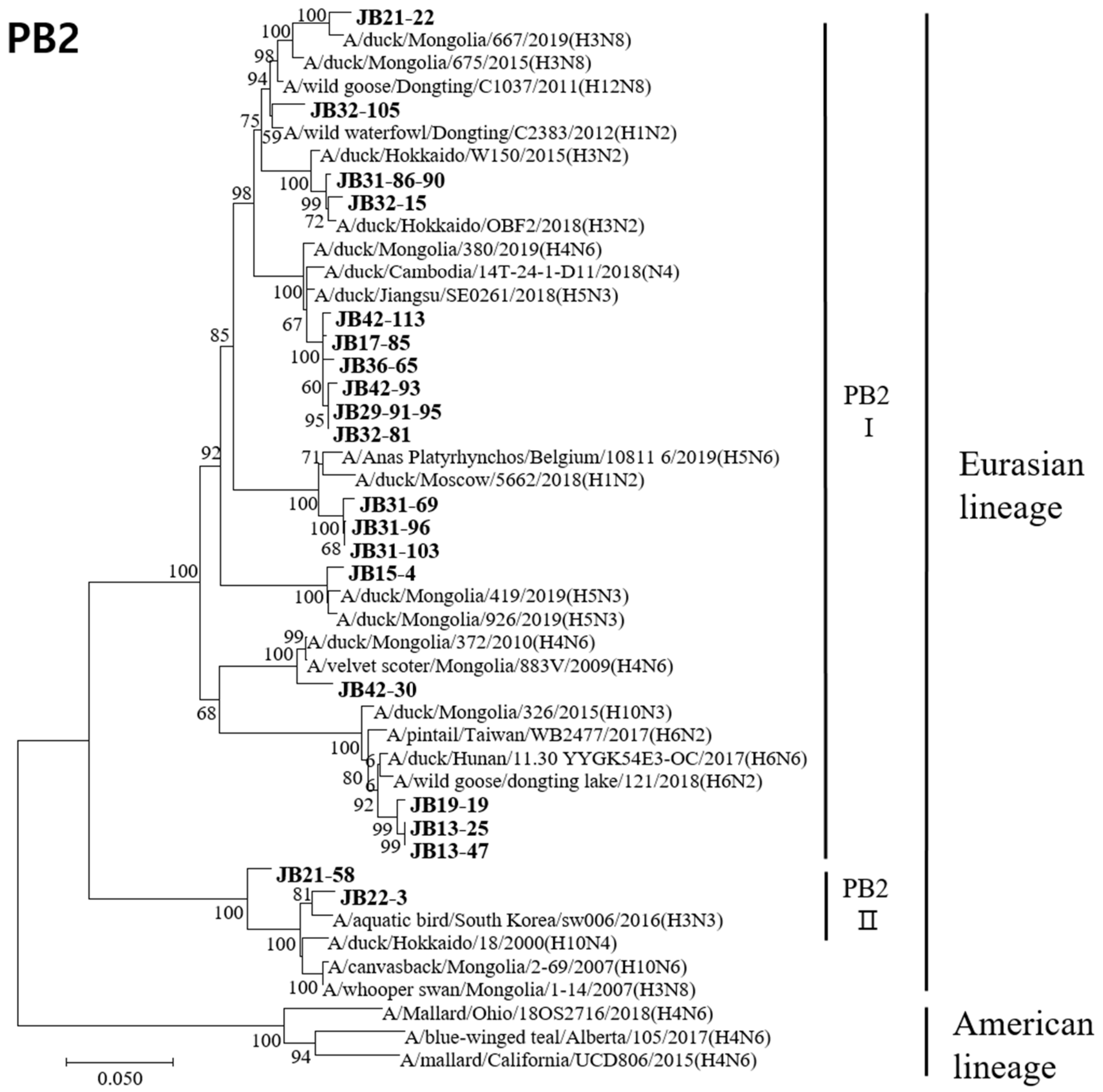

Supplementary Figure S2. Phylogenetic trees based on the nucleotide sequences of PB2 (a), PB1 (b), PA (c), NP (d), M (e), and NS (f) genes. Trees were produced by the neighbor-joining method and bootstrapped with 1000 replicates. Branch lengths are numbers of nucleotide substitutions per site; only bootstrap values at or above 50% are shown. Isolates identified in this study are shown in bold.

(b)

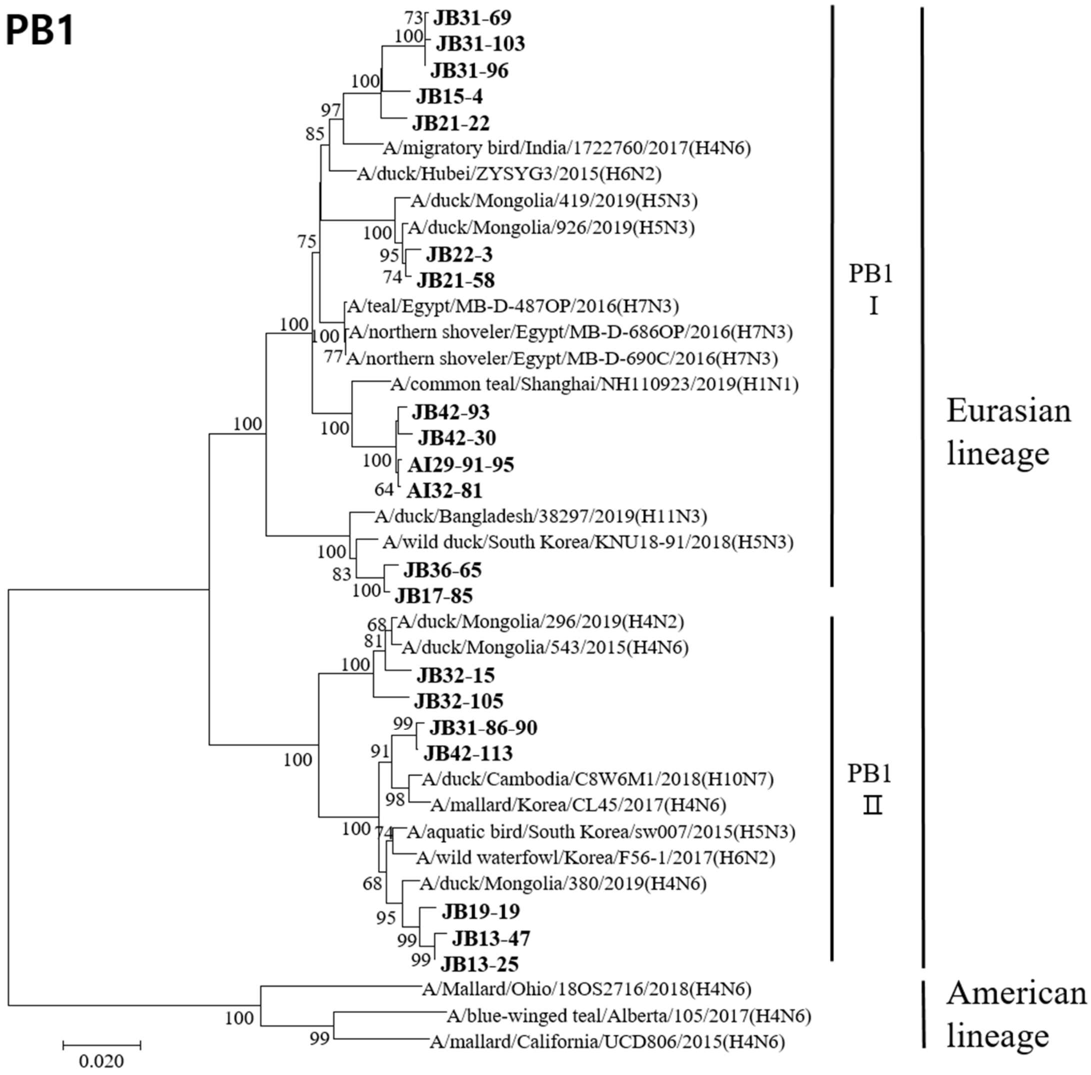

Supplementary Figure S2. *Cont.*

(c)

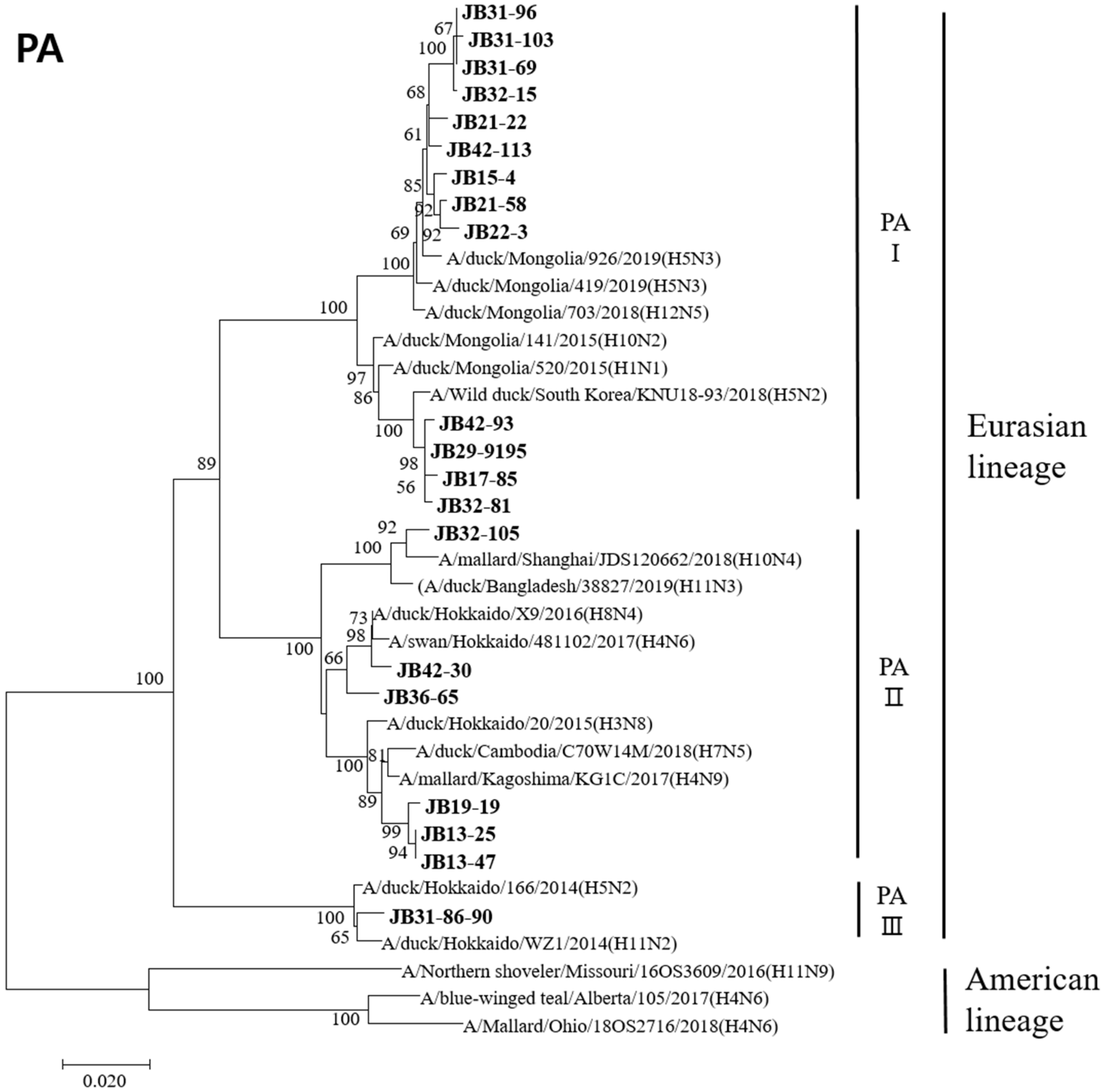

Supplementary Figure S2. *Cont.*

(d)

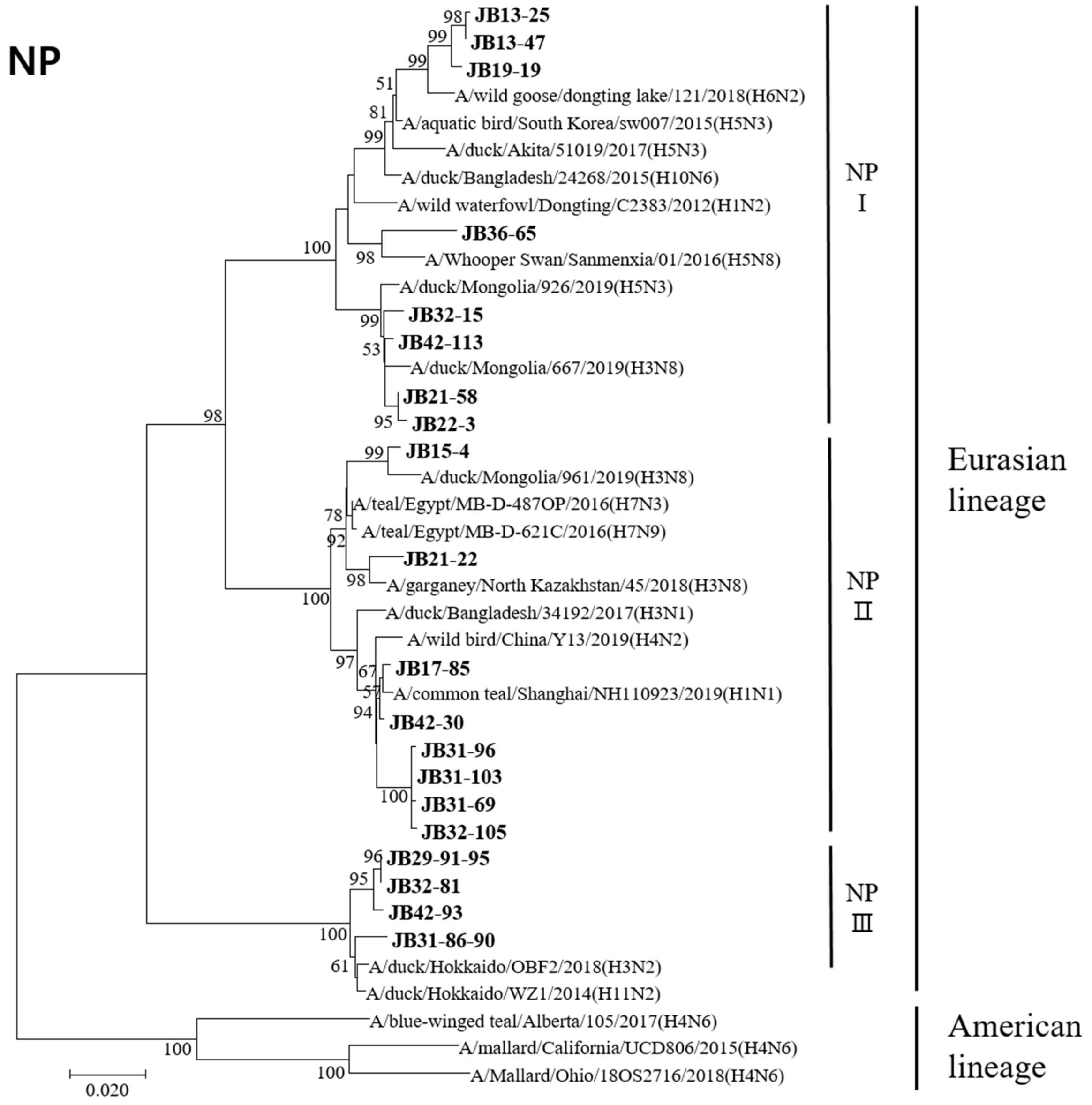

Supplementary Figure S2. *Cont.*

(e)

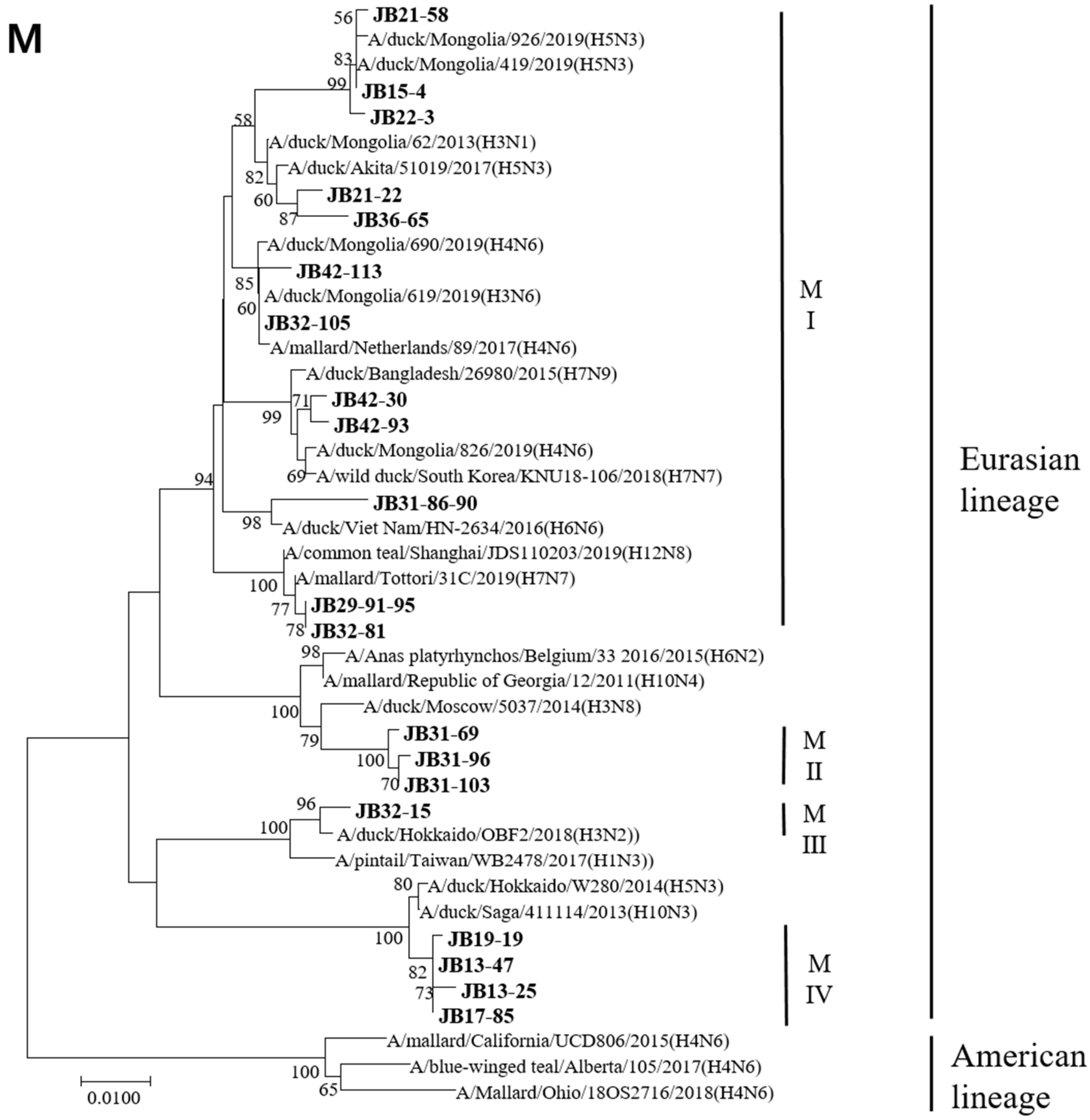

Supplementary Figure S2. *Cont.*

(f)

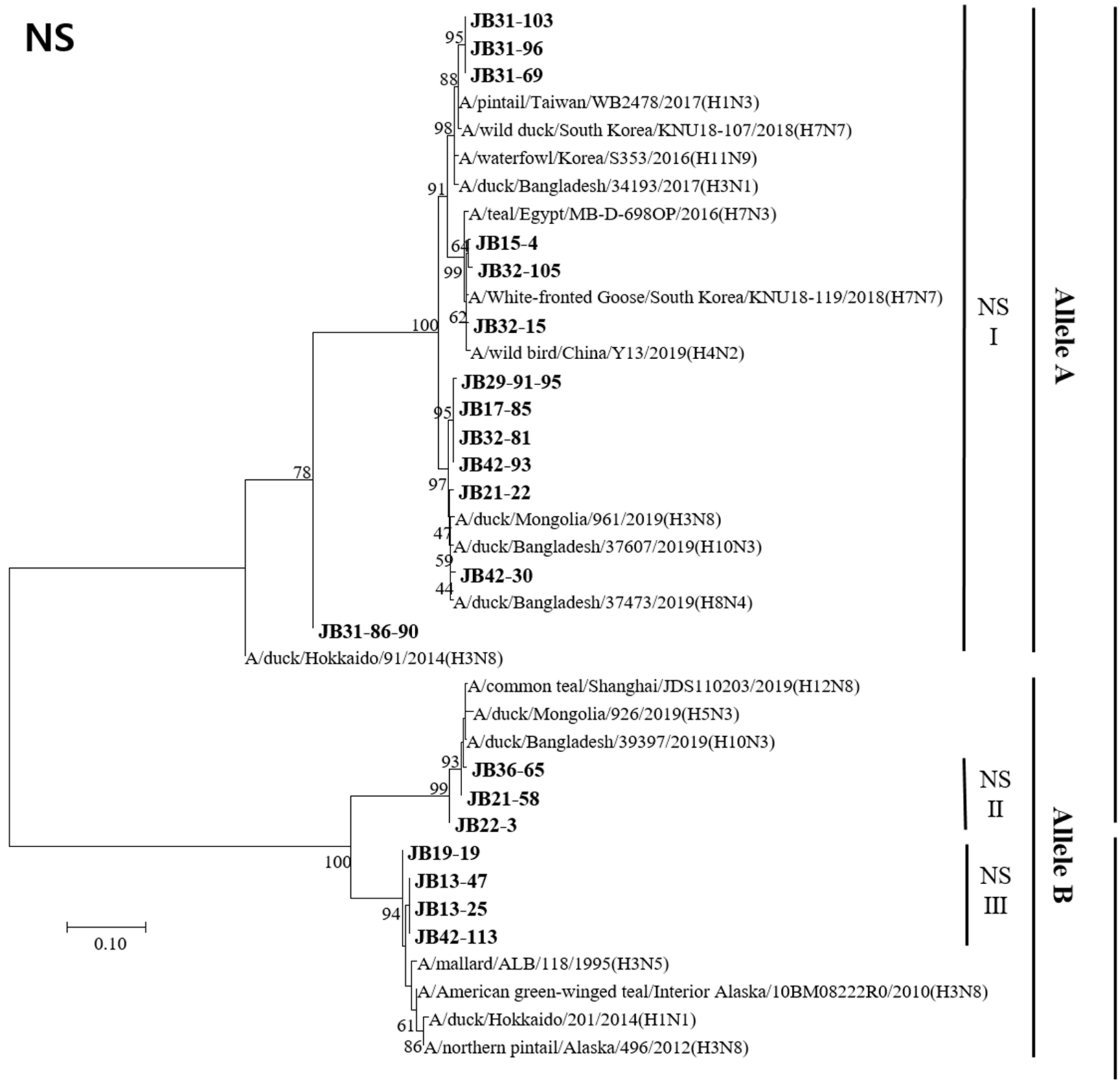

Supplementary Figure S2. *Cont.*

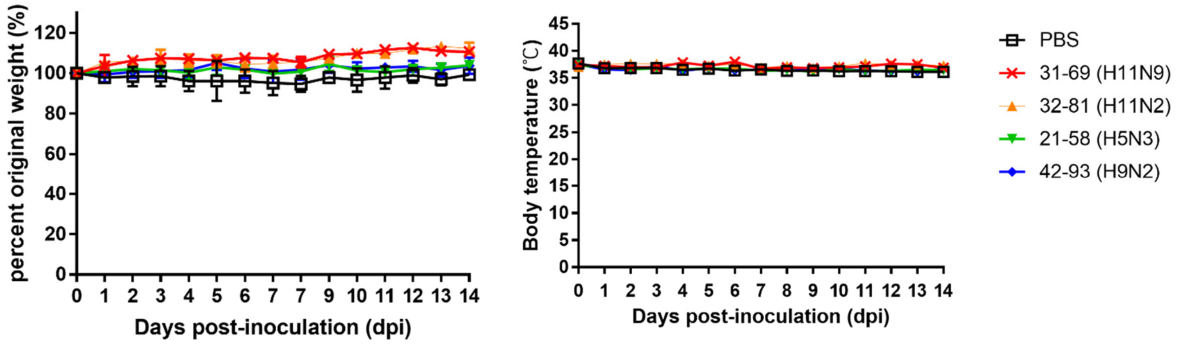

Supplementary Figure S3. Body weight (A) and body temperatures (b) of mice inoculated with JB31-69, JB32-81, JB21-58, JB42-93, and PBS. The mice were observed for 14 days post infection.

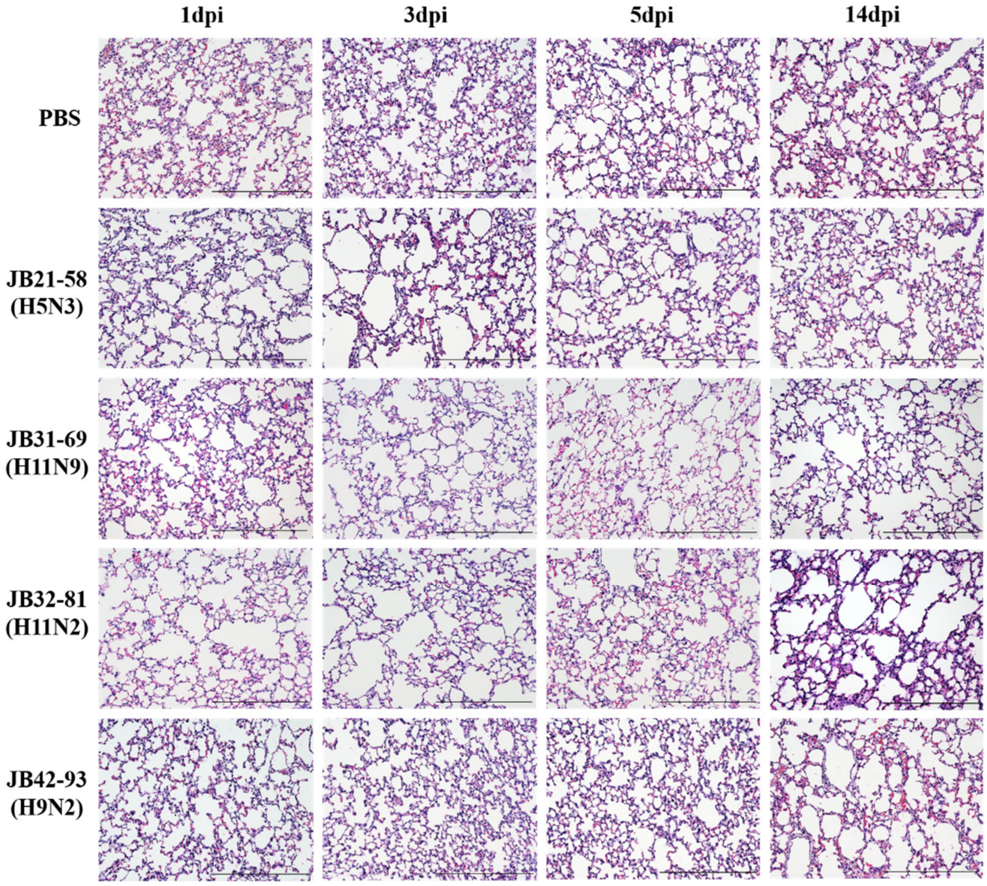

Supplementary Figure S4. Histopathological analysis of the lungs of mice inoculated with the four representative avian influenza viruses (AIVs): JB21-58 (H5N3), JB31-69 (H11N9), JB32-81 (H11N2), and JB42-93 (N9N2). The original magnification is x200, and the scale bar indicates 200 μm. Dpi: days post-infection.

Supplementary Table S1. The accession numbers in Genbank of isolated viruses.

Isolates	Abbreviation	Accession numbers in Gen Bank
A/spot-billed duck/South Korea/JB13-25/2019(H4N6)	JB13-25	MW485918-MW485924, MW486659
A/ spot-billed duck/ South Korea /JB13-47/2019(H4N6)	JB13-47	MW485873-MW485880
A/ spot-billed duck/ South Korea /JB15-4/2019(H4N3)	JB15-4	MW487380-MW487387
A/ mallard/ South Korea/JB17-85/2019(H4N6)	JB17-85	MW494137-MW494144
A/mallard/South Korea/JB19-19/2019(H6N8)	JB19-19	MW492914-MW492921
A/mallard/South Korea/JB21-22/2019(H4N6)	JB21-22	MW494129-MW494136
A/mallard/South Korea/JB21-58/2019(H5N3)	JB21-58	MW492924-MW492931
A/mallard/South Korea/JB22-3/2019(H5N3)	JB22-3	MW493128-MW493135
A/mallard/South Korea/JB29-91-95/2019(H11N2)	JB29-91-95	MW126486-MW126493
A/mallard/South Korea/JB31-69/2019(H11N9)	JB31-69	MW093394, MW093746-MW093752
A/spot-billed duck/South Korea/JB31-86-90/2019(H6N2)	JB31-86-90	MW493137-MW493144
A/mallard/South Korea/JB31-96/2019(H11N9)	JB31-96	MW116662-MW116669
A/mallard/South Korea/JB31-103/2019(H11N9)	JB31-103	MW116719-MW116726
A/Eurasian teal/South Korea/JB32-15/2019(H10N7)	JB32-15	MW494145-MW494152
A/spot-billed duck/South Korea/JB32-81/2019(H11N2)	JB32-81	MW116734-MW116741
A/spot-billed duck/South Korea/JB32-105/2019(H11N2)	JB32-105	MW494116-MW494123
A/Bean goose/South Korea/JB36-65/2019(H10N4)	JB36-65	MW493149-MW493156
A/Falcated duck/South Korea/JB42-30/2020(H9N2)	JB42-30	MW493158-MW493165
A/Falcated duck/South Korea/JB42-93/2020(H9N2)	JB42-93	MW493166-MW493173
A/mallard/South Korea/JB42-113/2020(H4N6)	JB42-113	MW494153-MW494160
